# Predictors of Mobile Health App Acceptance Among Nurses in Kashan, Iran: Cross-Sectional Study

**DOI:** 10.2196/93888

**Published:** 2026-07-31

**Authors:** Narjes Mirabootalebi, Felix Holl, Walter Swoboda, Zahra Tagharrobi, Fatemeh Rangraz Jeddi, Hossein Akbari

**Affiliations:** 1 Social Determinants in Health Promotion Research Center, Hormozgan Health Institute, Hormozgan University of Medical Sciences, Bandar Abbas, Iran; 2 Health Information Management Research Center, Kashan University of Medical Sciences, Kashan, Iran; 3 DigiHealth Institute, Neu-Ulm University of Applied Sciences, Neu-Ulm, Germany; 4 Leibniz Science Campus Digital Public Health, Bremen, Germany; 5 Trauma Nursing Research Center, Kashan University of Medical Sciences, 5th of Qotb-e Ravandi Blvd, Kashan, 8715973474, Iran, 98 (913) 1613899, 98 (913) 1613899; 6 Department of Health Information Management and Technology, Allied Medical Sciences Faculty, Kashan University of Medical Sciences, Kashan, Iran; 7 Social Determinants of Health (SDH) Research Center, Kashan University of Medical Sciences, Kashan, Iran

**Keywords:** mobile apps, acceptance, nurses, attitude, Iran

## Abstract

**Background:**

The integration of mobile health apps (MHAs) into nursing practice is essential for improving efficiency, conserving resources, and enhancing patient care. To promote the acceptance of these apps in clinical care, it is important to assess current acceptance levels and identify influencing factors.

**Objective:**

This study investigated the acceptance of MHAs and related factors among nurses in hospitals affiliated with Kashan University of Medical Sciences, Iran.

**Methods:**

In this cross-sectional study, 250 nurses were selected via stratified random sampling in Kashan in 2022. Data were collected using the Nurses’ Mobile Health Device Acceptance Scale (NMHDA-S) and the Probable Predictors Questionnaire. The data were analyzed using SPSS (version 16; IBM Corp), 1-way ANOVA, independent *t* test, Pearson correlation coefficients, and multiple linear regression.

**Results:**

The findings showed that nurses had a mean acceptance score of 4.207 (SD 0.740) on a Likert scale from 1 to 7 (95% CI 4.115‐4.299) regarding the use of MHAs. Multiple linear regression analysis indicated that 4 variables significantly predicted acceptance of MHAs (*R*²=0.230, *F*_4,245_=18.308, *P*<.001): interest in participating in relevant educational programs (β=0.392, *P*<.001), encouragement from health care professionals to use MHAs (β=0.133, *P*=.02), male participant (β=−0.116, *P*=.04), and duration of daily internet use (β=0.117, *P*=.04).

**Conclusions:**

Although MHA acceptance among clinical nurses in Kashan is moderate, adoption can be enhanced through targeted training, facilitated internet access, and gender-specific incentive policies, particularly for female staff.

## Introduction

The increasing prevalence of smartphones and mobile health apps (MHAs) among health care professionals highlights the growing integration of health information technology into clinical practice [1]. Nurses are encouraged to incorporate various forms of health information technology into their clinical practice for several reasons [2]. One primary reason is the nature of the nursing profession, which requires unrestricted mobility within the clinical setting [3]. On the other hand, the global shortage of nurses is a major challenge [4]. A study conducted in 2020 revealed that most nurses (52.4%) had a negative attitude toward adopting and using MHAs [5]. In addition, a study conducted in Iran [6] reported that approximately 63.4% of nurses did not use MHAs on their smartphones.

The absence of MHAs in the clinical setting can lead to several challenges associated with the use of paper. These challenges include significant time consumption, frequent visits to nursing stations for recording and retrieving information, and an increased risk of forgetting important details. Moreover, the use of paper increases the likelihood of errors in data recording and management, which may have serious consequences for patient safety. In this context, implementing MHAs at the patient’s bedside is essential and represents an inevitable solution. These technologies have led to fundamental improvements in the quality of nursing care and are straightforward and easily accessible [7]. Therefore, health care managers across various levels need to adopt MHAs to increase the quality-of-care services and address the issues arising from traditional paper-based systems. The initial step in addressing this challenge involves assessing the current acceptance of MHAs among nurses and identifying the factors that influence this acceptance [8].

The concept of acceptance concerning technologies such as MHAs is framed primarily through behavioral models, with the expectation-confirmation model (ECM) serving as the foundation for this study. To capture the unique aspects of the nursing context thoroughly, our ECM framework intentionally incorporates established components from the technology acceptance model and the unified theory of acceptance and use of technology (UTAUT). These include constructs such as perceived ease of use and social influence. This integrated theoretical approach operationalizes acceptance by measuring a range of multidimensional factors, thus ensuring that the model is directly aligned with the clinical environment, including effectiveness, usage, performance, and anxiety related to new technology [9,10].

Numerous studies have explored the factors influencing the acceptance of MHAs. However, a notable limitation of these studies is the inconsistent prioritization of identified factors among health care professionals [8,11,12]. For example, Lwoga and Lwoga [13] and Chopdar et al [14] emphasized perceived security risk, whereas Lau et al [15] reported no significant correlation between this factor and the acceptance of MHAs. Sezgin et al [16] emphasized the characteristics of the user interface as an important factor influencing the use of MHAs. However, the results of Hsiao and Chen [17] revealed that this factor does not have a significant influence. In addition, there is a discrepancy in the literature regarding the influence of organizational characteristics on the use of MHAs. While Zhou et al [18] demonstrated a significant effect, Hsiao and Chen [19] noted that this factor was not significant.

Despite the expanding body of literature on MHAS acceptance, most studies have been conducted in developed countries with well-established health information infrastructures. In contrast, developing nations such as Iran face distinct infrastructural, sociocultural, and economic challenges during the digital transformation of their health care systems. Kashan, a prominent center for medical education and clinical care in central Iran, offers an ideal environment for investigating these transitional dynamics. The 5 hospitals affiliated with Kashan University of Medical Sciences include a wide range of clinical wards and diverse nursing demographics, making them an ideal setting for a comprehensive examination of digital health adoption in a resource-limited environment. Given inconsistencies in the existing literature on the impact of attitudes on performance and the lack of comprehensive studies in such contexts, this research was conducted to assess the acceptability of using MHAs and related factors among clinical nurses across all 5 hospitals in Kashan, Iran. The results of this study can serve as a foundation for developing educational interventions and policy decisions to advance the implementation of digital health in nursing practice.

## Methods

### Study Design and Setting

The present study is a descriptive cross-sectional study. In this study, the sample size was calculated on the basis of a pilot study conducted with 30 nurses, which produced a mean acceptance score of 4.055 (SD 0.722). With a confidence level of 95% and an accuracy estimate (*d*) defined as a conservative fraction of the SD (*d* = 0.124 × 0.722), consistent with established methodological guidelines for achieving high precision in continuous data [20,21], Cochran formula was used to calculate the required sample size, resulting in a total of 250 participants (Equation 1).

The inclusion criteria included Iranian citizenship, a university degree in nursing, no known psychological disorders based on self-reports (such as mood disorders or anxiety disorders), at least 6 months of clinical experience, current employment in clinical roles in inpatient departments, and consent to participate in the study. Refusal to complete the data collection instruments was also considered as an exclusion criterion. A stratified random sample by department was conducted among eligible nurses in different clinical wards of hospitals affiliated with Kashan University of Medical Sciences. This sampling process was conducted over 2 months, from August to October 2022.


$$n=\frac{z^{2}\times {SD}^{2}}{d^{2}}$$

Equation 1 shows the Cochrane formula for sample size.

### Data Collection Process

After approval from the Research Council and the Ethics Committee of Kashan University of Medical Sciences, the first author of the article approached the nursing offices of 5 hospitals (Shahid Beheshti, Naghavi, Matini, Kargarnejad, and Seyed Al Shohada Hospitals) with an introductory letter. After the study procedure was explained and permission from hospital management was secured, a comprehensive list was compiled detailing all clinical departments, including the names and total numbers of active nursing staff in each unit. To ensure proportional representation in line with the inclusion criteria and the target sample size, a proportionate number of eligible nurses from each department was then selected via simple random sampling. After consent was obtained from the head nurses, the first author of the article visited the departments at the beginning of each shift in accordance with the work schedules of the selected nurses. After providing consent, the selected nurses were instructed to complete the data collection instruments. The instruments were collected at the end of each shift. If the scales were not completed within the specified time frame, an agreement was reached with the respective nurse regarding the time of submission. In cases where the selected sample was unavailable or uncooperative, another nurse from the same department was randomly selected. All data collection instruments were completed by the nurses themselves and were based on self-reports.

### Data Collection Instruments

In the present study, data were collected via the probable predictors of acceptance of MHAs by nurses using the Nurses’ Mobile Health Device Acceptance Scale (NMHDA-S) [8] (Multimedia Appendix 1).

The probable predictors of acceptance of mobile health programs, according to the nurses’ questionnaire, are divided into 4 sections:

1. Personal and professional information, including age, gender, marital status, education level, department, work experience, frequent shift work, having young or adolescent children, and holding a managerial position
2. Knowledge of MHAs, assessed via a single self-reported item rated on a 7-point scale, specifically asking: “Please indicate your level of knowledge regarding MHAs on a scale of 1 to 7”
3. A history of familiarity with MHAs, including observations of health care professionals or acquaintances using MHAs, participation in relevant courses, specialized university training, encouragement to use MHAs by health care professionals, involvement in relevant research, mandatory use of specific MHAs in the workplace, instances of patient inquiries about MHAs, the presence of individuals with educational or professional backgrounds in information technology or related fields among first-degree relatives or close friends, and their willingness to engage with topics about new technologies
4. Use of MHAs, including professional and personal use, smartphone ownership, duration of smartphone usage, device operating system, types of devices used, duration of daily internet use, and interest in participating in relevant educational programs

This questionnaire was developed in Persian by the researchers of the present article on the basis of a comprehensive literature review, including relevant articles and questionnaires, as well as a survey of nursing and health information management professionals. The qualitative content validity was confirmed by 10 faculty members from Kashan University of Medical Sciences. The reliability of the questionnaire was evaluated via the test-retest method with a group of 20 nurses over a 1-week interval. The degree of agreement between the 2 measurements was determined by calculating the intraclass correlation coefficient for quantitative variables (such as knowledge of MHAs) and the κ statistic for categorical variables (such as familiarity factors with MHAs). Notably, all the responses regarding personal and professional variables remained consistent across both assessments. The intraclass correlation coefficients for the quantitative variables were found to exceed 0.95, whereas the κ statistics for the categorical variables surpassed 0.97.

The NMHDA-S was used to evaluate the acceptance of MHAs among nurses. This scale was originally developed in Persian on the basis of the constructs of the ECM and was psychometrically evaluated by Mirabootalebi et al [8] in 2024. The NMHDA-S consists of 33 items categorized into 5 subscales: usage and performance (10 items), social influence (7 items), perceived ease of use (5 items), effectiveness (7 items), and new technology anxiety (4 items). The respondents rated each item on a 7-point Likert-type scale. To determine the total acceptance score, the mean of all the items is calculated, resulting in a continuous score ranging from 1 to 7, where higher values indicate greater acceptance. Subscale scores are derived via the same mean-based procedure. The scale’s construct validity has been confirmed, and it demonstrates high internal consistency, with Cronbach α and McDonald ω coefficients of 0.938 and 0.953, respectively. These indices were estimated to be above 0.88 for all the subscales [8].

### Data Analysis

The data were analyzed using SPSS version 16. Acceptance scores were rated on a 7-point scale (1 to 7), and the 95% CI was calculated for the target population. Skewness and kurtosis indices were used to assess the data distribution, with a range of ±2 indicating a normal distribution. To prioritize the scale factors, a stepwise multiple linear regression analysis was performed to determine the contributions of various factors to acceptance, including usage and performance, social influence, perceived ease of use, effectiveness, and new technology anxiety.

The effects of the factors were analyzed in 2 phases to identify predictors. In the first phase, univariate analyses, including independent *t* tests, one-way ANOVA, and Pearson correlation coefficients, were conducted to assess the relationship between each factor and MHA acceptance. Variables with a significance level of <0.20 were subsequently entered into a stepwise multiple linear regression model. To ensure the validity of the regression results, key assumptions were rigorously evaluated. Multicollinearity was assessed using variance inflation factors (VIFs) and tolerance indices, while autocorrelation among the residuals was examined using the Durbin-Watson statistic. The normality and homoscedasticity of the residuals were evaluated using objective statistical measures—specifically, skewness and kurtosis values within ±2 were considered indicative of normal distributions. This analysis was further supported by visual inspection of residual histograms and normal probability (P-P) plots. For all analyses, a *P* value of <.05 was considered statistically significant.

### Ethical Considerations

All necessary approvals were obtained from the Research Council (number 039, dated 2022) and the Ethics Committee of Kashan University of Medical Sciences (code 40138). The first author explained the study to each nurse individually in a quiet, private, and comfortable room within the department. The key points discussed included the purpose of the study, the expected actions required for participation, the estimated time needed to complete the instruments, measures to ensure the confidentiality of the data, the absence of negative consequences if they choose not to participate in the study, the individuals and departments overseeing the study, and their right to withdraw from participation at any time. After these explanations, each nurse provided verbal consent and then signed a written informed consent form.

## Results

### Participant Characteristics

Among the 306 eligible nurses, 250 (81.7%) participated in the study, resulting in the analysis of 250 samples. The mean age of the participants was 35.624 (SD 7.582) years. Table 1 presents the personal and professional information, knowledge of MHAs, history of familiarity with MHAs, and usage of MHAs. The mean knowledge score of MHAs was calculated to be 2.976 (SD 1.210) on a 7-point scale (1 to 7).

**Table 1.** Characteristics of the participants, Kashan, 2022 (N=250).

Variables	Values
Personal and professional information	
Gender, n (%)	
Man	44 (17.6)
Woman	206 (82.4)
Marital status, n (%)	
Married	201 (80.4)
Single	47 (18.8)
Divorced	2 (0.8)
Education, n (%)	
Bachelor	217 (86.8)
Master	33 (13.2)
Department, n (%)	
Emergency	23 (9.2)
Internal	31 (12.4)
Surgery	50 (20)
Intensive unit	51 (20.4)
Pediatrics	7 (2.8)
Operating room	34 (13.6)
Other	54 (21.6)
Frequent shift work, n (%)	
Morning	114 (45.6)
Evening	29 (11.6)
Night	36 (14.4)
Rotating	71 (28.4)
Having young or adolescent children, n (%)	
Yes	102 (40.8)
No	148 (59.2)
Having managerial position, n (%)	
Yes	49 (19.6)
No	201 (80.4)
Age (y), mean (SD)	35.624 (7.582)
Work experience (y), mean (SD)	11.594 (6.722)
History of familiarity with MHAs^a^	
Observations of health care professionals or acquaintances using MHAs, n (%)	
Yes	91 (36.4)
No	159 (63.6)
Participation in relevant courses, n (%)	
Yes	67 (26.8)
No	183 (73.2)
Specialized university training, n (%)	
Yes	47 (18.8)
No	203 (81.2)
Encouragement to use MHAs by health care professionals, n (%)	
Yes	57 (22.8)
No	193 (77.2)
Involvement in relevant research, n (%)	
Yes	27 (10.8)
No	223 (89.2)
Mandatory use of specific MHAs in the workplace, n (%)	
Yes	51 (20.4)
No	199 (79.6)
Instances of patient inquiries about MHAs, n (%)	
Yes	60 (24)
No	190 (76)
Presence of individuals with professional backgrounds in information technology or related fields among first-degree relatives or close friends, n (%)	
Yes	69 (27.6)
No	181 (72.4)
Willingness to engage with topics about new technologies [1-5], mean (SD)	3.184 (0.985)
Duration of smartphone usage (y), (n=231), mean (SD)	9.446 (3.340)
Interest in participating in relevant educational programs [1-5], mean (SD)	3.036 (0.937)
Use of MHAs, n (%)	
Professional use	
Yes	65 (26)
No	185 (74)
Personal use	
Yes	73 (29.2)
No	177 (70.8)
Smartphone ownership	
Yes	231 (92.4)
No	19 (7.6)
Device operating system (n=231)	
Android	207 (82.8)
Apple iOS	24 (9.6)
Type of devices used	
iPhone	19 (7.6)
Mobile phone	224 (89.6)
Mobile phone and tablet	7 (2.8)
Duration of daily internet use	
Less than 1 hour	49 (19.6)
1 to 2 hours	91 (36.4)
2 to 4 hours	62 (24.8)
More than 4 hours	48 (19.2)
NMHDA-S^b^ score (total and subscales), mean (SD)	
Usage and performance (on a scale of 1 to 7)	4.401 (0.952)
Social influence (on a scale of 1 to 7)	4.211 (1.219)
Perceived ease of use (on a scale of 1 to 7)	4.351 (1.384)
Effectiveness (on a scale of 1 to 7)	4.471 (1.185)
New technology anxiety (on a scale of 1 to 7)	3.602 (1.246)
Total score of NMHDA-S (on a scale of 1 to 7)	4.207 (0.740)

a MHA: mobile health app.

b NMHDA-S: Nurses' Mobile Health Device Acceptance Scale.

### Acceptance Levels

The mean acceptance score for using MHAs in nursing was 4.207 (SD 0.740; on a scale of 1 to 7), indicating a moderate level of acceptance among clinical nurses. This rate was estimated to be 4.207 (95% CI 4.115-4.299) within the target population. All quantitative variables in the current study had a normal distribution based on the criteria included in the methodology (skewness and kurtosis within ±2). Stepwise multiple linear regression was used to prioritize the subscales of the acceptance scale. The results revealed that the contributions of each subscale, including usage and performance, social influence, perceived ease of use, effectiveness, and new technology anxiety, to explaining the variations in acceptance ratings were 1.9%, 5.5%, 12.6%, 76.9%, and 3.1%, respectively. Consequently, effectiveness proved to be the most important subscale.

### Predictors of Acceptance

A significant univariate association was found between acceptance scores and several factors, including gender (*P*=.03), education level (*P*=.001), observations of health care professionals or acquaintances using MHAs (*P*<.001), participation in relevant courses (*P*=.002), encouragement to use MHAs by health care professionals (*P*<.001), instances of patient inquiries about MHAs (*P*=.002), the presence of individuals with educational or professional backgrounds in information technology or related fields among first-degree relatives or close friends (*P*<.001), professional (*P*=.001), and personal use (*P*=.03), duration of daily internet use (*P*=.009), interest in participating in relevant educational programs (*P*<.001), willingness to engage with topics about new technologies (*P*<.001), and knowledge of MHAs (*P*=.002; Tables 2 and 3).

**Table 2.** Acceptance scores for mobile health app (MHA) use in care according to categorical predictor variables among Iranian nurses, Kashan, 2022 (N=250).

Variable	Acceptance score, mean (SD)	*t* test (*df*) or *F* test (*df*)	*P* value
Gender		2.235^a^ (248)	.03
Woman	4.159 (0.723)		
Man	4.432 (0.783)		
Marital status		4.437^b^ (2, 2.863)	.13
Married	4.181 (6.990)		
Single	4.288 (0.899)		
Divorced	4.904 (0.305)		
Education		−3.368^a^ (248)	.001
Bachelor	4.147 (0.718)		
Master	4.603 (0.769)		
Department		0.795^c^ (6, 243)	.58
Emergency	4.047 (0.619)		
Internal	4.243 (0.861)		
Surgery	4.330 (0.702)		
Intensive unit	4.217 (0.675)		
Pediatrics	4.232 (0.421)		
Operating room	4.023 (0.719)		
Other	4.244 (0.846)		
Frequent shift work		1.786^c^ (3, 246)	.15
Morning	4.103 (0.760)		
Evening	4.172 (0.678)		
Night	4.290 (0.766)		
Rotating	4.347 (0.705)		
Having young or adolescent children		−0.735^a^ (248)	.44
Yes	4.166 (0.697)		
No	4.236 (0.769)		
Having managerial position		1.044^a^ (248)	.30
Yes	4.306 (0.716)		
No	4.183 (0.745)		
Observations of health care professionals or acquaintances using MHAs		3.860^a^ (248)	<.001
Yes	4.440 (0.741)		
No	4.074 (0.708)		
Participation in relevant courses		3.206^a^ (248)	.002
Yes	4.451 (0.768)		
No	4.118 (0.711)		
Specialized university training		0.944^a^ (248)	.35
Yes	4.311 (0.866)		
No	4.183 (0.708)		
Encouragement to use MHAs by health care professionals		3.700^a^ (248)	<.001
Yes	4.518 (0.770)		
No	4.115 (0.707)		
Involvement in relevant research		0.822^a^ (248)	.42
Yes	4.350 (0.981)		
No	4.190 (0.706)		
Mandatory use of specific MHAs in the workplace		0.610^a^ (248)	.54
Yes	4.264 (0.742)		
No	4.193 (0.741)		
Instances of patient inquiries about MHAs		3.195^a^ (248)	.002
Yes	4.468 (0.752)		
No	4.125 (0.718)		
Presence of individuals with professional backgrounds in IT or related fields among first-degree relatives or close friends		3.606^a^ (248)	<.001
Yes	4.474 (0.700)		
No	4.105 (0.731)		
Professional use		3.414^a^ (248)	.001
Yes	4.710 (0.767)		
No	4.114 (0.709)		
Personal use		2.229^a^ (248)	.03
Yes	4.368 (0.808)		
No	4.141 (0.701)		
Smartphone ownership		1.783^a^ (248)	.08
Yes	4.231 (0.752)		
No	3.918 (0.504)		
Device operating system		0.803^a^ (248)	.42
Android	4.245 (0.757)		
iOS	4.114 (0.710)		
Type of devices used		1.661^b^ (2, 13.303)	.23
iPhone	4.367 (0.423)		
Mobile	4.181 (0.728)		
Mobile and tablet	4.626 (1.479)		
Duration of daily internet use (h)^d^^,^^e^		3.975^c^ (3, 246)	.009
<1 (A)	3.948 (0.676)		
1‐2 (B)	4.275 (0.708)		
2‐4 (C)	4.142 (0.760)		
>4 (D)	4.427 (0.769)		

a Independent *t* test.

b ANOVA (Welch statistic).

c ANOVA (*F* statistic).

d Pairwise comparison: A and B: *P*=.06; A and C: *P*=.65; A and D: *P*=.008; B and C: *P*=.84; B and D: *P*=.80; D and C: *P*=.23.

e Post hoc (Gabriel).

**Table 3.** Correlation of acceptance scores for using mobile health apps (MHAs) in care with quantitative variables among Iranian nurses, Kashan, 2022 (N=250).

Variable	*r* values^a^	*P* value
Age (y)	–0.01	.87
Work experience (y)	0.052	.41
Duration of smartphone usage (y; n=231)	0.128	.04
Interest in participating in relevant educational programs [1-5]	0.419	<.001
Willingness to engage with topics about new technologies [1-5]	0.276	<.001
Knowledge of MHAs [1-7]	0.196	.002

a Pearson coefficient.

A stepwise multiple linear regression was performed to further examine the relationships between the independent variables and acceptance scores. The VIFs for all the variables were <5, and their tolerances exceeded 0.10, indicating an absence of multicollinearity. The Durbin-Watson statistic was 1.943, which falls within the acceptable range of 1.5 to 2.5. Before conducting the final regression analysis, the underlying assumptions were thoroughly verified. The Durbin-Watson statistic was within the acceptable range, confirming the absence of autocorrelation. The VIF values indicated no multicollinearity. For the regression residuals, both skewness and kurtosis were within the acceptable range of ±2, suggesting normality. Additionally, examination of residual histograms and normal P-P plots further confirmed that the assumptions of normality and homoscedasticity were fully satisfied. Consequently, the assumptions of multiple linear regression were satisfied, confirming the validity of the model. The results show that the simultaneous inclusion of 4 variables in the model is significant (*F*_4,245_=18.308, *P*<.001) and explains 23% of the observed variance in acceptance scores. These variables include interest in participating in relevant educational programs, encouragement to use MHAs by health care professionals, gender, and duration of daily internet use. The variable with the greatest influence is interest in participating in relevant educational programs, which accounts for 17.6% of the observed changes. Interest in participating in relevant educational programs, encouragement by health care professionals to use MHAs, and duration of daily internet use positively influence acceptance. In addition, being a man is associated with greater acceptance (Table 4).

**Table 4.** Results of multiple linear regression to determine the predictive factors influencing the acceptance of using mobile health apps (MHAs) among Iranian nurses.

Model^a^	*R* ^2^	B^b^ (95% CI)	SE	β^c^	*t* value	*P* value	Collinearity statistics	
							Tolerance	VIF^d^
Constant	—^e^	3.886 (3.202 to 4.570)	0.347	—	11.184	<.001	—	—
Interest in participating in relevant educational programs (scoring 1‐5)	0.176	0.310 (0.221 to 0.398)	0.045	0.392	6.881	<.001	.966	1.035
Encouragement to use MHAs by health care professionals^f^	0.024	0.235 (0.035 to 0.435)	0.102	0.133	2.310	.02	.943	1.060
Gender^g^	0.017	−0.226 (−0.442 to −0.010)	0.110	−0.116	−2.059	.04	.981	1.019
Duration of daily internet use (h)	0.013	0.086 (0.003 to 0.168)	0.042	0.117	2.049	.04	.961	1.041

a *R*2=0.230, *F*_4,245_=18.308, *P*<.001.

b Unstandardized coefficients.

c Standardized coefficients.

d VIF: variance inflation factors.

e Not applicable.

f Reference group=no.

g Reference group=man.

When controlling for other variables, the average acceptance score for MHAs among female nurses was 0.226 points lower than that of male nurses. In contrast, nurses who received encouragement from health care professionals to use MHAs experienced an average increase of 0.235 points in their acceptance scores. For each 1-unit increase in interest in participating in related educational programs, the acceptance score increased by an average of 0.310 points. Additionally, each extra hour of daily internet usage corresponds to an average increase of 0.086 points in the acceptance score. In this model, the unstandardized coefficients (B) represent the absolute change in the dependent variable per unit change in the independent variable, measured in the original units of the independent variable. The standardized coefficients (β) are dimensionless values that normalize the variables by their SDs, allowing them to be assessed on a common scale. While B quantifies the absolute magnitude of change, β is used to interpret the relative importance and compare the predictive power of the independent variables, regardless of their measurement units. According to the β coefficients in Table 4, interest in participating in relevant educational programs has the highest relative explanatory power in the model.

## Discussion

### Acceptance of MHAs Among Nurses

The present study aimed to investigate the acceptance of MHAs and associated factors among clinical nurses in Iran. The principal findings indicate moderate acceptance of MHAs among the surveyed nurses, with a mean score of 4.207 (SD 0.740) out of 7.

A study conducted by Shanmugapriya et al [22] in India reported that 71.6% of nursing students strongly agreed, 27.8% agreed, and only 0.6% expressed no opinion regarding their acceptance of MHAs. In addition, Kürtüncü et al [7] reported a mean total score of 124.69 (SD 15.98; ranging from 0‐128) for Turkish nurses on the MHAS acceptance model, indicating a high level of acceptance in this group. The researchers explained that the reason for the high level of acceptance among Turkish nurses was related to their high use of smartphones [7]. In Iran, the acceptance of MHAs among nurses was found to be moderate, possibly due to the lack of necessary infrastructure, absence of established guidelines, and insufficient knowledge [6]. In simpler terms, nurses are still not sufficiently equipped for digital transformation in health care and face several challenges, including limited digital skills, restrictions on smartphone usage, and financial constraints. By examining these specific behavioral factors in a resource-limited context such as Iran, this study goes beyond incremental findings to provide a comprehensive understanding of the unique challenges of digital health adoption in developing health care systems. It is recommended that incentives be offered to nurses to help offset the costs of using their personal smartphones for clinical tasks [23].

The findings of the current study suggest that the dimensions of “effectiveness” and “perceived ease of use” contribute significantly to the variations in acceptance scores. In contrast, “usage and performance” was responsible for the least variation in these scores. These results are consistent with those of previous studies. Hsiao and Chen [19] highlighted the impact of maturity and perceived usefulness on technology acceptance. Recent evidence on the adoption of health care technology indicates that perceived usefulness and perceived ease of use play significant roles in technology acceptance and the intention to use these innovations [24]. Additionally, recent studies focusing on nurses and health care professionals emphasize that perceived usefulness is an essential factor influencing both technology acceptance and the intention to use it [24,25]. In a review paper, Nezamdoust et al [26] identified 8 key themes influencing nurses’ use of MHAs: ease of use, usefulness, security and privacy, feasibility and performance, design and user interface, effectiveness, infrastructure, adaptability, and social norms. The findings of this and other studies indicate that perceived usefulness, perceived ease of use, and maturity are important determinants of clinical nurses’ acceptance of MHAs. Owing to living in the information and technology era and recognizing the benefits of using MHAs, nurses are increasingly inclined to use similar systems. Therefore, it is clear that perceived ease of use and usefulness play important roles in the acceptance of MHAs and ultimately encourage their increased or continued use.

In the present study, “usage and performance” made the smallest contribution to explaining the difference in caregiver acceptance of MHAs. Consistent with these findings, Hsiao and Chen [19] concluded that individual habits do not significantly influence the acceptance or performance of MHAs. In addition, Sezgin et al [16] reported no significant relationships between outcome acceptance, habits, self-efficacy, expected performance, creativity, and community influence on behavioral intentions. Chen et al [27] also concluded that security risk has a negative effect on the intention to use. However, some studies support the rejection of the hypothesis of the present study. Recent findings indicate that privacy and performance risks negatively impact trust and the intention to adopt digital health technologies, suggesting that perceived risk reduces acceptance [24]. Studies by Lwoga and Lwoga [13] and also Chopdar et al [14] have emphasized the influence of perceived security risk. The factors influencing usage and performance include personal habits, security risk, and social approval. The underutilization of MHAs can be attributed to several challenges, such as the absence of robust policies, lack of practical and user-friendly apps, and nurses’ unawareness of the potential benefits of MHAs. These issues likely play an important role in the resulting usage scores.

On the basis of the results of the present study, “interest in participating in relevant educational programs,” “encouragement to use MHAs by health care professionals,” “gender,” and “duration of internet use per day” were identified as factors influencing nurses’ acceptance of using MHAs in their nursing practice. The greatest contribution was “interest in participating in relevant educational programs,” which was significantly positively correlated with the acceptance rate.

Kaya et al [28] reported a significant association between in-service training and changes in nurses’ attitudes toward themselves, computers, and information technology devices. This technology requires training and attention to organizational and environmental factors to increase technology anxiety among nurses. In addition, it is important to create a supportive organizational culture that promotes technical skills and provides adequate time and resources for learning [29,30]. In a 2019 study [31], 69.5% of nurses could not use technology effectively and correctly. One of the main reasons for this is nurses’ lack of computer skills. Addressing this issue through continuous education for nurses can help bridge the generational and educational divide, ultimately alleviating their anxiety about using new technologies. Lifelong education for nurses plays a critical role in improving their acceptance of technology, especially in the face of constant changes in patient care, advancements in knowledge, and evolving practices and policies. In addition, supervisor and peer support can encourage nurses to embrace information technology by actively promoting MHA, effectively integrating it into clinical practice, and recognizing the resulting performance improvements.

The present study revealed that encouraging the use of MHAs among health care professionals has a positive and significant effect on acceptance rates. Recent evidence indicates that social factors significantly influence users’ intention to adopt technology, whereas attitudes remain a key contributor in these contexts [32]. Recent studies have shown that social influence significantly predicts the intention to use emerging technologies [33]. The findings of the study by Alasmari and Zhang [34] are consistent with those of the present study. The acceptance of MHAs by nurses is significantly influenced by various factors, including use by nursing colleagues, other health care professionals, and care managers. For example, colleagues who integrate smartphones into their clinical practice can be role models for nurses and encourage them to use similar technologies. In addition, the positive experiences and satisfaction of colleagues and managers with MHAs can help reduce nurses’ technology-related anxiety and reservations and thus increase their overall acceptance of these tools. In particular, sharing positive experiences with experts can be a strong motivator for nurses. When selecting MHAs, nurses usually consult more experienced colleagues, often giving considerable weight to their recommendations. Consequently, a positive view from peers can significantly increase nurses’ acceptance of technologies. When colleagues endorse specific technologies, this promotes a sense of approval among health care professionals. Conversely, peer resistance to MHAs can have a negative effect on acceptance and usage.

In the statistical model outlined in the present study, the “duration of daily internet usage” is found to have a positive effect on the acceptance score. Shanmugapriya et al [22] reported that there is a statistically significant correlation between the acceptance of MHAs and the time spent on nursing studies (internet-based learning). A 2020 study [35] identified several significant predictors of acceptance of MHAs among patients with cancer, including age, education level, and access to the internet. In addition, the duration of daily internet use increased among nurses using smartphones, which in turn impacted their acceptance of these apps. Smartphones provide easy access to the internet and allow nurses to access important online resources, such as treatment and medication guidelines, directly from their workplace. This capability enhances the ease of access and utilization of digital information in the clinical setting. In addition, smartphones facilitate communication with colleagues, physicians, and patients via various platforms, such as email, messaging applications, and telemedicine services.

This study revealed that men had a greater level of acceptance. A 2019 study [34] revealed that men tended to use mobile learning technologies more strongly than women did. In addition, a study by Nunes et al [36] confirmed the significant influence of men on the acceptance of MHAs. In this context, Al-Azawei [37] pointed to gender difference as a moderator in the relationship between the model constructs. However, in this study, this moderating effect was found to be minimal with respect to the relationship between e-learning self-efficacy and learning management system acceptance. Self-efficacy had a stronger effect on the intention to use learning management system in men than in women, suggesting that men have relatively higher levels of acceptance [37]. However, a 2024 study [38] examining students’ intentions to use online learning through educational technology applications based on the UTAUT model revealed a significant influence of women on acceptance. Recent evidence suggests that both gender and age can affect the relationship between self-efficacy and technology adoption, with self-efficacy remaining a crucial factor in users’ intentions to adopt technology [39]. Furthermore, the findings indicate that specific computer effectiveness has a more pronounced effect on perceived ease of use than does general remaining efficacy. Among cofactors, general computer self-efficacy emerged as a significant predictor of perceived usefulness for both men and women. Additionally, while perceptions of general computer self-efficacy were lower, it still had a notable influence on the intention to use technology. Conversely, specific computer self-efficacy demonstrated a stronger indirect effect on behavioral intentions among women, although both genders reported similar levels of self-efficacy [40]. The results can serve as a reference for hospitals and clinical training organizations. When integrating nursing technologies into nurses’ clinical practice, it is important to consider user characteristics and relevant moderators when specific groups are targeted through MHAs. Studies suggest that men are more at ease with and acclimatized to technology than women are. In addition, societal expectations around gender roles can influence how people perceive and interact with technology. In a country with cultural characteristics similar to those of Iran, it seems that women’s greater anxiety about new technology and men’s greater belief in their self-efficacy contribute to these results. Further research is needed, as there is a lack of robust evidence in this area for different societies. Analyzing the underlying causes in Iranian society highlights the need for qualitative approaches.

Importantly, several factors may contribute to the discrepancies between the results of the current study and those of previous related research. These factors include the timing and location of the studies, the type of statistical tests, the presence or absence of established national guidelines, differences in the relevant target groups, and the specific characteristics of those populations.

The substantial sample size and the ability to conduct multiple analyses are notable strengths of the current study. Several factors may play important roles for health care professionals, including task mobility, individual performance, technological maturity [19], self-efficacy, responsiveness [41], organizational support, environmental and organizational influences, adaptability [17], and acceptance of MHA; however, these factors were not explored in the current study. In this context, the final multiple linear regression model (Table 4) explained only 23% of the variance in the acceptance score, indicating that 77% of the variance is explained by other variables not included in the model. Consequently, the interpretation of these results should be approached with more caution, as the identified predictors provide only a partial explanation of MHAS acceptance. Furthermore, the timing of sampling during the COVID-19 crisis disrupted the usual structure of hospital departments, changed the composition of human resources, and significantly increased the occupational stress among nurses in this setting [42]. As a result, the unwillingness of a considerable number of nurses to participate in the study probably affected the generalizability of the findings, which is recognized as a limitation of this study. It is recommended that a study be conducted under normal conditions, free from the constraints imposed by pandemic crises, to address the limitations of the current study. In addition, conducting a qualitative study with a grounded theory approach would be valuable for an in-depth investigation of the factors influencing the acceptance of MHAs by nurses in different societies, including Iran.

This study has several limitations. First, the data were collected concurrently with the COVID-19 crisis, during which the nursing staff experienced a high workload and occupational stress [42], potentially affecting participation and responses. A substantial proportion of eligible nurses were unwilling to cooperate, which may have introduced response bias. Furthermore, the participants’ knowledge of MHAs was assessed using a single-item question, which may not have captured the full multidimensionality of this construct. It is strongly recommended that future studies evaluate this variable via more appropriate, comprehensive, and highly precise measurement tools.

### Conclusions

The present study revealed moderate acceptance of MHAs among nurses in hospitals affiliated with Kashan University of Medical Sciences. The key factors that positively and significantly contribute to the acceptance rate include “interest in participating in relevant educational programs,” “encouragement from health care professionals to use MHAs,” and “duration of daily internet use.” In addition, the acceptance rate is significantly higher among men than women. Facilitating interest in participation in relevant training courses, alongside the provision of adequate internet access and the implementation of incentive policies targeting all nursing professionals, especially women, may increase the acceptance of MHAs in the delivery of care. Therefore, health policy makers and nursing managers should recognize the importance of this issue when designing continuing education programs for nurses, formulating policies related to incentive structures, and establishing supportive infrastructure in hospitals. By implementing effective strategies, they can improve the acceptance of MHAs among nurses in the clinical setting.

## Supplementary material

10.2196/93888 Multimedia Appendix 1 Data collection instruments (in Persian).

## Abbreviations

ECM: expectation-confirmation model
MHA: mobile health app
NMHDA-S: Nurses’ Mobile Health Device Acceptance Scale
VIF: variance inflation factor
