# Supplementary material for: Predictors of Mobile Health App Acceptance Among Nurses in Kashan, Iran: Cross-Sectional Study
Source: JMIR Mhealth Uhealth. 2026 Jul 31;14:e93888. doi: 10.2196/93888 (PMC13426121; doi:10.2196/93888)
Supplement: Multimedia Appendix 1 [file mhealth-v14-e93888-s001.docx]

**Multimedia Appendix 1. Data Collection Instruments**

**به نام خدا**

پرستار گرامی، این پرسشنامه با هدف طراحی و روانسنجی پرسشنامه نگرش پرستاران نسبت به استفاده از برنامه‌های کاربردی مبتنی بر تلفن همراه در عملکرد پرستاری بر اساس تئوری انتظار-تائيد تدوین گردیده است.

لطفاً به دقت به کلیه­ی سؤالات پاسخ دهید.

**پرسشنامه عوامل زمینه­ای و پیشگویی­کننده­های احتمالی استفاده از برنامه‌های سلامت همراه**

**اطلاعات شخصی شغلی**

1) سن: .......... سال

2) جنس: الف) مذکر ⬜ ب) مؤنث ⬜

3) وضعیت تأهل: الف) متأهل ⬜ ب) مجرد ⬜ ج) مطلقه ⬜ د) بیوه ⬜

4) تحصیلات: الف) کارشناسی ⬜ ب) کارشناسی ارشد ⬜ ج) دکتری ⬜

5) نوع بخش بالینی: الف) اورژانس ⬜ ب) بخش­های داخلی ⬜ ج) جراحی ⬜ د) بخش­های مراقبت ویژه ⬜ ه) اطفال ⬜ و) روان ⬜ ز) گوش، حلق، بینی ⬜ ح) اتاق عمل ⬜ ط) ساير (نام ببريد) ⬜ : ..........

6) میزان سابقه کاری: .......... سال

7) شیفت کاری غالب: الف) صبح ⬜ ب) عصر ⬜ ج) شب ⬜

8) داشتن فرزندی در سنين نوجوانی يا جوانی: الف) بلی ⬜ ب) خیر ⬜

9) داشتن سمت در سطوح مديريت پرستاری (مترون، سوپروايزر، سرپرستار): الف) بلی ⬜ ب) خیر ⬜

**سطح آگاهی در رابطه با برنامه‌های کاربردی سلامت همراه**

10) سطح آگاهی خود را از برنامه‌های کاربردی سلامت همراه در مقياس 1 تا 7 مشخص سازيد؟ 1) خیلی ضعیف ⬜ 2) ضعیف ⬜ 3) متوسط ⬜ 4) خوب ⬜ 5) خیلی خوب ⬜ 6) عالی ⬜ 7) خیلی عالی ⬜

**زمینه آشنایی با برنامه‌های کاربردی سلامت همراه**

11) آيا تا به حال شاهد استفاده از برنامه‌های سلامت همراه توسط اعضاء تيم مراقبت سلامت يا اطرافيان خود بوده‌ايد؟ الف) بلی ⬜ ب) خير ⬜

12) آيا تا به حال در دوره­های آموزشی مرتبط شرکت نموده­ايد؟ الف) بلی ⬜ ب) خیر ⬜

13) آيا در دوران تحصيل، آموزش اختصاصی دريافت کرده­ايد؟ الف) بلی ⬜ ب) خیر ⬜

14) آيا تا به حال توسط عضوی از تيم مراقبت سلامت به استفاده از برنامه‌های سلامت همراه ترغيب شده‌ايد؟ الف) بلی ⬜ ب) خیر ⬜

15) آيا تا به حال در انجام پژوهشی مرتبط مشارکت داشته‌ايد؟ الف) بلی ⬜ ب) خیر ⬜

16) آيا در بخش محل اشتغال، الزامی در رابطه با به‌کارگیری نوعی از برنامه‌های کاربردی سلامت همراه وجود داشته يا دارد؟ الف) بلی ⬜ ب) خیر ⬜

17) آيا تا به حال با سؤال يا درخواستی از طرف بيماران در رابطه با استفاده از برنامه‌های سلامت همراه مواجه شده‌ايد؟ الف) بلی ⬜ ب) خير ⬜

18) آيا در اقوام درجه يک شما (همسر، فرزند، پدر، مادر، خواهر و يا برادر) فردی با زمينه تحصيلی يا شغلی مرتبط با رشته فناوری اطلاعات يا ساير علوم وابسته وجود دارد؟ الف) بلی ⬜ ب) خير ⬜

19) ميزان تمايل خود را به دنبال کردن مباحث مربوط به استفاده از تکنولوژی نوين مشخص سازید؟ 0) بی‌علاقه ⬜ 1) اندک ⬜ 2) نسبی ⬜ 3) زياد ⬜ 4) خيلی زياد ⬜

**پرسشنامه وضعيت استفاده از ابزار و برنامه‌های کاربردی سلامت همراه**

**1) آيا تا به حال از برنامه‌های کاربردی سلامت همراه در مراقبت از بيمارانتان و يا ساير ابعاد مرتبط با عملکرد حرفه‌اي خود استفاده کردید؟**

بلی ⬜ خیر ⬜

1-1) در صورت انتخاب گزينه بلی، ميزان استفاده خود را در مقياس 1 تا 10 (1 نشان‌دهنده کمترين ميزان استفاده و 10 بيانگر بيشترين ميزان است) مشخص سازيد؟ ..........

1-2) در صورت انتخاب گزينه بلی، نوع دستگاه یا دستگاه‌های مورد استفاده را نام ببريد (آیفون، آيپد، تبلت، تلفن همراه و ...): ..........

1-3) در صورت انتخاب گزينه بلی، نوع برنامه یا برنامه‌های کاربردی را نام ببريد: ...........

1-4) در صورت انتخاب گزينه بلی، هدف یا اهداف استفاده را مشخص سازيد: ...........

1-5) در صورت انتخاب گزينه بلی، ميزان رضايت از استفاده را مشخص سازيد:

0) ناراضی ⬜ 1) رضايت اندک ⬜ 2) رضايت نسبی ⬜ 3) رضايت زياد ⬜ 4) رضايت خيلی زياد ⬜

**2) آيا تا به حال از برنامه‌های کاربردی سلامت همراه در زندگی شخصی خود استفاده کردید؟**

بلی ⬜ خیر ⬜

2-1) در صورت انتخاب گزينه بلی، ميزان استفاده خود را در مقياس 1 تا 10 (1 نشان‌دهنده کمترين ميزان استفاده و 10 بيانگر بيشترين ميزان است) مشخص سازيد؟ ..........

2-2) در صورت انتخاب گزينه بلی، نوع دستگاه یا دستگاه‌های مورد استفاده را نام ببريد (آیفون، آيپد، تبلت، تلفن همراه و ...): ..........

2-3) در صورت انتخاب گزينه بلی، نوع برنامه یا برنامه‌های کاربردی مورد استفاده را نام ببريد: ..........

2-4) در صورت انتخاب گزينه بلی، هدف یا اهداف استفاده را مشخص سازيد: ..........

2-5) در صورت انتخاب گزينه بلی، ميزان مفيد بودن آن را مشخص سازيد:

0) بی‌فایده ⬜ 1) اندک ⬜ 2) متوسط ⬜ 3) زياد ⬜ 4) خيلی زياد ⬜

**3) آیا شما تلفن همراه هوشمند دارید؟**

بله ⬜ خیر ⬜

3-1) در صورت انتخاب گزينه بلی، چند سال است که از تلفن همراه هوشمند استفاده می‌کنید؟ ..........

3-2) در صورت انتخاب گزينه بلی، دستگاه تلفن همراه چه سیستم عاملی دارد؟

اندروید ⬜ Apple IOS ⬜ ویندوز ⬜

**4) کدام دستگاه یا دستگاه‌ها را در اختيار داريد (مي‌توانيد بيش از يک گزينه انتخاب کنيد)؟**

آیفون ⬜ آیپد ⬜ تلفن همراه ⬜ تبلت ⬜ سایر (نام ببريد) ⬜: ..........

**5) در طول روز چه مدت از اينترنت استفاده مي‌کنيد؟**

کمتر از 1 ساعت ⬜ 1 تا 2 ساعت ⬜ 2 تا 4 ساعت ⬜ بیشتر از 4 ساعت ⬜

**6) مهم‌ترین عامل تأثیرگذار روی استفاده از برنامه‌های سلامت همراه کدام مورد است؟**

دوستان ⬜ خانواده ⬜ اينترنت ⬜ رسانه‌های گروهی (راديو و تلويزيون) ⬜ ژورنال‌های تخصصی ⬜ پرسنل بهداشتی درمانی ⬜ اطلاعيه‌های فروشگاه‌های مرتبط ⬜ ساير (لطفاً مشخص نمایید) ⬜: ..........

**7) ميزان علاقه خود را به شرکت در برنامه‌های آموزشی مرتبط در مقياس صفر تا چهار مشخص نمایید:**

صفر (بی‌علاقه) ⬜ 1 (علاقه کم) ⬜ 2 (علاقه نسبی) ⬜ 3 (علاقه زياد) ⬜ 4 (خيلی علاقه‌مند) ⬜

**8) تا چه حد استفاده از برنامه‌های کاربردی سلامت همراه را به بيمارانتان پيشنهاد مي‌کنيد (در مقياس صفر تا چهار مشخص نمایید):**

صفر (اصلاً) ⬜ 1 (کم) ⬜ 2 (در حد متوسط) ⬜ 3 (زياد) ⬜ 4 (خيلی زياد) ⬜

**9) به نظر شما در استفاده از برنامه‌های کاربردی سلامت همراه در عملکرد پرستاری هر يک از عوامل زير تا چه حد به‌عنوان بازدارنده نقش دارند؟**

⬜ نگرانی در مورد امنيت اطلاعات بيمار:

صفر (اصلاً) ⬜ 1 (کم) ⬜ 2 (در حد متوسط) ⬜ 3 (زياد) ⬜ 4 (خيلی زياد) ⬜

⬜ در اختيار نداشتن دستگاه مورد نياز:

صفر (اصلاً) ⬜ 1 (کم) ⬜ 2 (در حد متوسط) ⬜ 3 (زياد) ⬜ 4 (خيلی زياد) ⬜

⬜ ناکارآمدی برنامه‌های موجود:

صفر (اصلاً) ⬜ 1 (کم) ⬜ 2 (در حد متوسط) ⬜ 3 (زياد) ⬜ 4 (خيلی زياد) ⬜

⬜ عدم طراحی برنامه‌های مناسب و متناسب با نياز حرفه‌ای پرستاران:

صفر (اصلاً) ⬜ 1 (کم) ⬜ 2 (در حد متوسط) ⬜ 3 (زياد) ⬜ 4 (خيلی زياد) ⬜

⬜ عدم آگاهی از بهترين برنامه‌های طراحی‌شده:

صفر (اصلاً) ⬜ 1 (کم) ⬜ 2 (در حد متوسط) ⬜ 3 (زياد) ⬜ 4 (خيلی زياد) ⬜

⬜ نداشتن انگيزه و علاقه:

صفر (اصلاً) ⬜ 1 (کم) ⬜ 2 (در حد متوسط) ⬜ 3 (زياد) ⬜ 4 (خيلی زياد) ⬜

⬜ عدم پذيرش فناوری مربوطه توسط بيماران:

صفر (اصلاً) ⬜ 1 (کم) ⬜ 2 (در حد متوسط) ⬜ 3 (زياد) ⬜ 4 (خيلی زياد) ⬜

⬜ زمان‌بر بودن آشنايي با فناوری مربوطه و کسب مهارت در زمينه به‌کارگیری:

صفر (اصلاً) ⬜ 1 (کم) ⬜ 2 (در حد متوسط) ⬜ 3 (زياد) ⬜ 4 (خيلی زياد) ⬜

⬜ هزينه‌بر بودن استفاده از فناوری مربوطه:

صفر (اصلاً) ⬜ 1 (کم) ⬜ 2 (در حد متوسط) ⬜ 3 (زياد) ⬜ 4 (خيلی زياد) ⬜

⬜ ترديد در مورد قابل اعتماد بودن محتوای برنامه‌ها:

صفر (اصلاً) ⬜ 1 (کم) ⬜ 2 (در حد متوسط) ⬜ 3 (زياد) ⬜ 4 (خيلی زياد) ⬜

⬜ فقدان زيرساخت‌های لازم (مثل عدم دسترسی به شبکه وای فای، ....):

صفر (اصلاً) ⬜ 1 (کم) ⬜ 2 (در حد متوسط) ⬜ 3 (زياد) ⬜ 4 (خيلی زياد) ⬜

⬜ مهارت و تجربه ناکافی در استفاده:

صفر (اصلاً) ⬜ 1 (کم) ⬜ 2 (در حد متوسط) ⬜ 3 (زياد) ⬜ 4 (خيلی زياد) ⬜

⬜ ساير موارد (لطفاً مشخص نمایید): ..........

صفر (اصلاً) ⬜ 1 (کم) ⬜ 2 (در حد متوسط) ⬜ 3 (زياد) ⬜ 4 (خيلی زياد) ⬜

**پرسشنامه نگرش پرستاران نسبت به استفاده از برنامه‌های کاربردی سلامت همراه در عملکرد پرستاری**

| **گویه‌** | | **پاسخ** | | | | | | |
| --- | --- | --- | --- | --- | --- | --- | --- | --- |
|  |  | کاملاً مخالفم | مخالفم | تا حدودی مخالفم | نظری ندارم | تا حدودی موافقم | موافقم | کاملاً موافقم |
| **سهولت استفاده درک‌شده** | 1. پرستار مي‌تواند در مراقبت از بيمار، به آسانی از برنامه‌های کاربردی مبتنی بر تلفن همراه (app) استفاده نمايد. |  |  |  |  |  |  |  |
|  | 2. پرستار با استفاده از برنامه‌های مبتنی بر تلفن همراه می‌تواند فعالیت‌های مربوط به مراقبت از بیمار را راحت‌تر انجام دهد |  |  |  |  |  |  |  |
|  | 3. یادگیری نحوه استفاده از برنامه‌های کاربردی مبتنی بر تلفن همراه در ارائه مراقبت‌های پرستاری، آسان است. |  |  |  |  |  |  |  |
|  | 4. کسب مهارت در رابطه با استفاده از برنامه‌های کاربردی مبتنی بر تلفن همراه در ارائه مراقبت‌های پرستاری، به راحتی امکان‌پذير است. |  |  |  |  |  |  |  |
|  | 5. استفاده پرستار از برنامه‌های کاربردی مبتنی بر تلفن همراه در مراقبت از بيمار، به صرفه‌جويي در وقت کمک مي‌کند |  |  |  |  |  |  |  |
| **تأثیر اجتماع** | 6. مدیران پرستاری اعتقاد دارند که پرستاران بایستی از برنامه‌های کاربردی مبتنی بر تلفن همراه در مراقبت از بیمار استفاده کنند. |  |  |  |  |  |  |  |
|  | 7. سازمان‌های بالادستی نظير وزارت بهداشت، درمان و آموزش پزشکی، در به‌کارگیری برنامه‌های کاربردی مبتنی بر تلفن همراه توسط پرستاران نقش اساسی دارند. |  |  |  |  |  |  |  |
|  | 8. مدیران ارشد بیمارستان‌ها و دانشگاه‌ها از به‌کارگیری برنامه‌های کاربردی مبتنی بر تلفن همراه توسط پرستاران حمایت می‌کنند |  |  |  |  |  |  |  |
|  | 9. پرستاران استفاده از برنامه‌های کاربردی مبتنی بر تلفن همراه را در ارائه مراقبت‌های پرستاری به سایر همکاران خود توصيه نموده و بر آن تأکید دارند |  |  |  |  |  |  |  |
|  | 10. پزشکان از به‌کارگیری برنامه‌های کاربردی مبتنی بر تلفن همراه در ارائه مراقبت‌های پرستاری، استقبال مي‌کنند. |  |  |  |  |  |  |  |
|  | 11. پيام‌های ارسال‌شده از طريق رسانه‌های گروهی و شبکه‌های اجتماعی، پرستاران را به استفاده از برنامه‌های کاربردی مبتنی بر تلفن همراه در مراقبت از بيمار، ترغيب مي‌کنند |  |  |  |  |  |  |  |
| **اضطراب فناوری جدید** | 12. پرستاران به دلیل ترس ناشی از عدم امکان اصلاح خطا، نسبت به استفاده از برنامه‌های کاربردی مبتنی بر تلفن همراه در ارائه مراقبت‌های پرستاری، ترديد دارند. |  |  |  |  |  |  |  |
|  | 13. الزام استفاده از برنامه‌های کاربردی مبتنی بر تلفن همراه در ارائه مراقبت‌های پرستاری، باعث ترس و دلهره در پرستاران مي‌شود |  |  |  |  |  |  |  |
|  | 14. استفاده از برنامه‌های کاربردی مبتنی بر تلفن همراه در مراقبت از بیماران، برای پرستاران گیج‌کننده بوده و باعث سردرگمی آن‌ها مي‌شود. |  |  |  |  |  |  |  |
|  | 15. پرستاران هنگام استفاده از برنامه‌های کاربردی مبتنی بر تلفن همراه در مراقبت از بیماران، تنش زیادی را به دلیل عدم توانايي در مديريت مشکلات احتمالی تجربه مي‌کنند. |  |  |  |  |  |  |  |
| **عادت شخصی** | 16. استفاده از برنامه‌های کاربردی مبتنی بر تلفن همراه در مراقبت از بیمار، در بین پرستاران امری عادی و طبيعی محسوب می‌شود. |  |  |  |  |  |  |  |
|  | 17. پرستاران در مراقبت از بيماران ترجيح مي‌دهند از برنامه‌های کاربردی مبتنی بر تلفن همراه کمک بگيرند. |  |  |  |  |  |  |  |
|  | 18. پرستاران بايد برای ارائه مراقبت‌های پرستاری از برنامه‌های کاربردی مبتنی بر تلفن همراه استفاده کنند. |  |  |  |  |  |  |  |
| **ریسک امنیتی ادراک‌شده** | 19. هر چقدر پرستاران از امنیت اطلاعات شخصی بیماران در هنگام استفاده از برنامه‌های کاربردی مبتنی بر تلفن همراه برای ارائه مراقبت‌های پرستاری، اطمينان بيشتری داشته‌ باشند، از آن برنامه‌ها بیشتر استفاده می‌کنند |  |  |  |  |  |  |  |
|  | 20. پرستاران در صورتی تمایل به استفاده از برنامه‌های کاربردی مبتنی بر تلفن همراه برای ارائه مراقبت‌ دارند که مطمئن باشند داده‌های مربوط به بیمار و يا بیمارستان در دسترس افراد غیرمجاز قرار نمی‌گیرد. |  |  |  |  |  |  |  |
|  | 21. استفاده از برنامه‌های کاربردی مبتنی بر تلفن همراه در ارائه مراقبت‌های پرستاری، خطر احتمالی دست‌کاری داده‌های مربوط به بیماران و يا بیمارستان توسط افراد غیرمجاز را افزايش مي‌دهد. |  |  |  |  |  |  |  |
| **تأیید** | 22. استفاده از برنامه‌های کاربردی مبتنی بر تلفن همراه در ارائه مراقبت‌های پرستاری، بیش از سطح انتظار پرستاران، به ارتقاء کیفیت مراقبت کمک می‌کند |  |  |  |  |  |  |  |
|  | 23. استفاده از برنامه‌های کاربردی مبتنی بر تلفن همراه در ارائه مراقبت‌های پرستاری، بیش از سطح انتظار پرستاران، به ايجاد هماهنگی بين اعضاء تيم درمان در پردازش اطلاعات بيمار و تصميم‌گيری مناسب، کمک می‌کند. |  |  |  |  |  |  |  |
|  | 24. استفاده از برنامه‌های کاربردی مبتنی بر تلفن همراه در ارائه مراقبت‌های پرستاری، بیش از سطح انتظار پرستاران، انجام مداخلات درمانی و مراقبتی را سرعت مي‌بخشد. |  |  |  |  |  |  |  |
|  | 25. استفاده از برنامه‌های کاربردی مبتنی بر تلفن همراه در ارائه مراقبت‌های پرستاری، بیش از سطح انتظار پرستاران، فرايند مديريت خدمات پرستاری را بهبود مي‌بخشد |  |  |  |  |  |  |  |
|  | 26. استفاده از برنامه‌های کاربردی مبتنی بر تلفن همراه در ارائه مراقبت‌های پرستاری، بیش از سطح انتظار پرستاران، به اجرای مناسب و مؤثر دستورالعمل‌ها و راهنماهای بالینی مراقبت از بیمار کمک می‌کند. |  |  |  |  |  |  |  |
| **بلوغ** | 27. پرستاران برای انجام وظايف اصلی و اساسی محوله، مي‌توانند از برنامه‌های کاربردی مبتنی بر تلفن همراه استفاده‌ ‌کنند. |  |  |  |  |  |  |  |
|  | 28. قابلیت‌های برنامه‌های کاربردی مبتنی بر تلفن همراه با عملکرد بالینی پرستاران قابل انطباق و سازگار است |  |  |  |  |  |  |  |
|  | 29. برنامه‌های کاربردی مبتنی بر تلفن همراه برای کمک به انجام فعاليت‌های روزمره پرستاران، از کفايت لازم برخوردار می‌باشند |  |  |  |  |  |  |  |
| **سودمندی درک‌شده** | 30. استفاده از برنامه‌های کاربردی مبتنی بر تلفن همراه در ارائه مراقبت‌های پرستاری، بهره‌وری پرستاران را افزايش مي‌دهد. |  |  |  |  |  |  |  |
|  | 31. استفاده از برنامه‌های کاربردی مبتنی بر تلفن همراه در ارائه مراقبت‌های پرستاری، فرايند جمع‌آوری، مستندسازی و تحلیل داده‌های بالینی بیماران را بهبود مي‌بخشد. |  |  |  |  |  |  |  |
|  | 32. استفاده از برنامه‌های کاربردی مبتنی بر تلفن همراه در انجام مراقبت‌های پرستاری، به ارائه مراقبت خانواده¬‌محور کمک کرده و دخالت مستقيم پرستاران در انجام برخی مداخلات نظير دارو دادن را کاهش مي‌دهد |  |  |  |  |  |  |  |
|  | 33. استفاده از برنامه‌های کاربردی مبتنی بر تلفن همراه در ارائه مراقبت‌های پرستاری، ارتباط پرستاران را با ساير اعضاء تيم مراقبت سلامت بهبود مي‌بخشد |  |  |  |  |  |  |  |
